# Indoor Positioning System Using Magnetic Field Map Navigation and an Encoder System

**DOI:** 10.3390/s17030651

**Published:** 2017-03-22

**Authors:** Han-Sol Kim, Woojin Seo, Kwang-Ryul Baek

**Affiliations:** School of Electronics Engineering, Pusan National University, Busan 46241, Korea; hsv138@pusan.ac.kr (H.-S.K.); seowoojin@pusan.ac.kr (W.S.)

**Keywords:** magnetic field map, particle filter, indoor navigation, absolute position estimation

## Abstract

In the indoor environment, variation of the magnetic field is caused by building structures, and magnetic field map navigation is based on this feature. In order to estimate position using this navigation, a three-axis magnetic field must be measured at every point to build a magnetic field map. After the magnetic field map is obtained, the position of the mobile robot can be estimated with a likelihood function whereby the measured magnetic field data and the magnetic field map are used. However, if only magnetic field map navigation is used, the estimated position can have large errors. In order to improve performance, we propose a particle filter system that integrates magnetic field map navigation and an encoder system. In this paper, multiple magnetic sensors and three magnetic field maps (a horizontal intensity map, a vertical intensity map, and a direction information map) are used to update the weights of particles. As a result, the proposed system estimates the position and orientation of a mobile robot more accurately than previous systems. Also, when the number of magnetic sensors increases, this paper shows that system performance improves. Finally, experiment results are shown from the proposed system that was implemented and evaluated.

## 1. Introduction

As the need for indoor navigation grows, various techniques for indoor navigation are rapidly developing. Many businesses provide customers with various services based on a positioning system for their own purposes. In particular, industrial mobile robots, such as auto-guided vehicles, are widely used, and the market is gradually increasing. Also, home appliance robots, like the robot cleaner, are supplied in homes. In order to use these services, a navigation system needs to know the absolute position of the robot or smart device.

Navigation systems are classified into two categories [1]. One category uses absolute positioning, and the other uses relative positioning. In general, absolute position is estimated using external devices, such as beacons, landmarks, etc. Outdoors, the global positioning system (GPS) is mostly used to estimate absolute position. Recently, GPS standard positioning services have provided 3.1 m horizontal accuracy at a 95% confidence level [2]. But the GPS has to use four or more satellites [3]. This system is not suitable indoors, because the GPS signal cannot reach there. In order to use an absolute positioning system indoors, various positioning systems, such as ultrasonic [1,4,5], Bluetooth [6,7], and Wi-Fi [8,9] positioning systems, have been developed. However, these systems must be installed in the area where the positioning system is serviced. The relative positioning system does not need infrastructure because relative positioning sensors, such as an encoder system and an inertial navigation system (INS) [10], are only installed in the mobile robot. Also, the output rate of these systems is higher than with an absolute positioning system. However, the relative positioning system has a divergence problem when the position is calculated, so these systems need to correct for position error [1].

Magnetic field map navigation is able to estimate absolute position without infrastructure and the divergence problem. Indoors, the geo-magnetic field is refracted by structures like pillars, steel structures, and fixed large objects [11,12]. As a result of this refraction, a magnetic field map can be obtained and used in various methods [13]. These methods have one thing in common: that is, the use of a likelihood function. Many likelihood techniques have been researched to estimate absolute position. There are three types of likelihood method [13]. The first is maximum likelihood estimation (MLE), which selects the position with the maximum value. The second method is maximum likelihood ratio (MLR), which estimates the position of a mobile robot by using the distance between the first maximum position and the second maximum position. If the distance is shorter than a threshold value, the first maximum position is selected. If not, the estimated position is ignored. Finally, there is the aggregate bin likelihood (ABL) method, which uses an n-by-n moving average mask (bin). After moving-average computation, the position with the maximum value is selected as the estimated position. Depending on the size of the mask, system performance is determined. Above all, if many likelihoods have a high value, these three methods may also select a position with a large position error from among many candidate positions. 

The methods based on a particle filter can estimate the optimal position, even though there are many candidate positions with a high value. The particle filter is one of the recursive Bayesian estimators, and is robust against nonlinear/non-Gaussian filtering problems [14,15]. Since the measurement probability density function (PDF) is non-Gaussian in magnetic field map navigation, a particle filter was used for this paper [13,16]. Described briefly, the particle filter, which is sequential importance resampling (SIR) in this system, can be divided into two steps [14]. The first step is importance sampling. The particles are propagated according to a dynamic model. Also, the weights of particles are updated by a measurement probability density function. The final step is resampling. In order to avoid the problem of degeneracy, the effective number of particles is calculated to determine whether to resample [14,15,16]. If the effective number is smaller than a threshold value, the particles are resampled. Finally, all steps are repeated. There are various methods based on the particle filter, depending on how to propagate the particles, how to make the measurement probability density function, and how to resample the particles. Self-localization magnetic field map methods based on a particle filter have been researched. These systems propagate the particles by specific situations, such as the speed limit, without any other positioning system in the propagation step [13,16]. However, if the mobile robot motion is outside a fixed specific situation, particle propagation is inaccurate and inefficient. A magnetic map navigation system combined with an INS was introduced [17]. The combination with the INS can make the system more stable than a self-localization system, because the INS is used to know the user’s forward velocity and angular velocity to propagate particles. However, the INS has a divergence problem due to cumulative error and velocity, and the angular velocity results of the INS need to be corrected by other means.

In order to solve these problems and to increase performance, we propose a particle filter system that integrates magnetic field map navigation and an encoder system. In the propagation step, the proposed system determines velocity by using encoders instead of the INS to avoid the divergence problem without error correction. In order to use the encoder, the error characteristics of the encoder must first be known. The encoder system has two categories of error [18,19]. One is systematic errors from unequal wheel diameters, misalignment of wheels, or limited encoder resolution and limited sampling rates. The other is non-systematic errors from travelling over uneven floors and from wheel-slippage. If these errors accumulate over time, the estimated position of the mobile robot can diverge from the real position [20,21]. However, the encoder system is free from the divergence problem when velocity is calculated because the current velocity of the encoder system is not affected by the previously measured velocity. Therefore, the particles can be propagated by using the velocity of the encoder system without encoder error correction. In the measurement update step, multiple magnetic sensors and three magnetic field maps (a horizontal intensity map, a vertical intensity map, and a direction information map) are used to make the measurement PDF, which update the weights of particles. Also, in this paper the mobile robot’s orientation is computed by using only the magnetic field map without any other sensor, such as the encoder or the INS.

The organization of this paper is as follows: The magnetic-field map-building system is introduced in Section 2.1, and the magnetic field maps are introduced in Section 2.2. The various coordinate systems are described in Section 2.3. In Section 2.4, magnetic field map navigation using the particle filter is discussed. Section 3 shows the results from evaluation of the proposed system. Finally, Section 4 offers conclusions.

## 2. Positioning System

### 2.1. The Magnetic-Field Map-Building System

**Figure 1:**
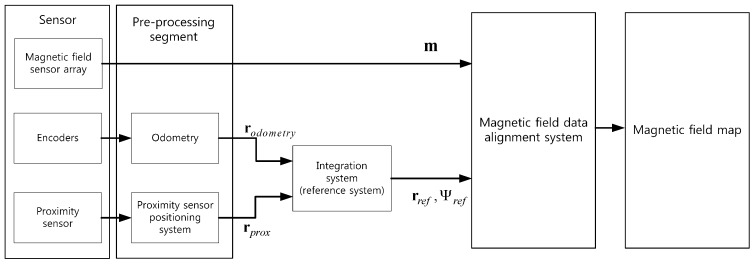
The magnetic-field map-building system.

In order to use magnetic field map navigation, a magnetic field map should be obtained in advance. Since the mobile robot moves with a constant height on an even floor, the magnetic field map should be built on the horizontal plane. In order to build the magnetic field map efficiently, a magnetic-field map-building system is needed. This system consists of a magnetic field sensor array, odometry, and a magnetic field data alignment system. When the system moves in the test area, the position of the system is estimated by odometry, and the magnetic field data is measured at the same time. Finally, the magnetic field map is built when the measured magnetic field data are aligned to position, estimated by odometry in the magnetic field data alignment system. In a previous study, an encoder system was used for odometry [22]. However, odometry using encoders has a divergence problem with estimated position $r_{odometry}$ because of cumulative error. Therefore, an integration system is needed to calibrate this error [20,21,22,23,24]. In order to calibrate the position error from odometry, we used an integration system with a capacitive binary proximity sensor, which is the PR30-10DN. Figure 1 shows the magnetic-field map-building system in this paper. This system consists of the sensor, a pre-processing segment, an integration system, and the magnetic field data alignment system. The sensors (the magnetic sensor array, two encoders, and a proximity sensor) are mounted in the mobile robot. The magnetic field data and the encoder data are measured at regular intervals. However, the proximity sensor does not output the signal at a regular time interval, because the proximity sensor generates a signal when the distance from a landmark that is installed at a known position in the test area is within 10 mm. The measured sensor data are processed during pre-processing, which consists of odometry and the proximity sensor positioning system. After pre-processing, $r_{odometry}$ from odometry and $r_{prox}$ from the proximity sensor positioning system are transmitted to the integration system. The estimated position, $r_{odometry}$ from odometry, is compensated for with the integration system when the new proximity sensor position data, $r_{prox}$, are received. Also, the this system calculates reference orientation $\Psi_{ref,k}$, which is expressed as

(1) $$\Psi_{ref,k}=\tan^{-1}\left(\frac{r_{ref,y,k}-r_{ref,y,k-1}}{r_{ref,x,k}-r_{ref,x,k-1}}\right)$$

where ${\left[r_{ref,x,k},r_{ref,y,k}\right]}^{T}=r_{ref,k}$, and *k* is the time index.

The maximum error from the proximity sensor is within 5 mm because the test area is not perfectly flat. However, this system has an acceptable level of error in the position estimating reference system of the mobile robot. Finally, a magnetic field map is built completely by the magnetic field data alignment system using the measured magnetic field data, m, reference position, $r_{ref}$, and reference orientation, $\Psi_{ref}$. Since the position of each magnetic sensor is known in mobile robot’s body frame, the position of each magnetic sensor in main frame is computed using reference position and orientation. The magnetic field measured at each sensor is first mapped to each sensor position in the main frame. Also, the measured X, Y direction magnetic field should be rotated by the reference orientation. Since the magnetic field map is constructed at constant interval, the magnetic field of each coordinate in the magnetic field map is computed by a bilinear interpolation about four reference positions near each coordinate. After interpolation, the magnetic field map is obtained using a 3 × 3 mean filter.

### 2.2. The Magnetic Field Map

**Figure 2:**
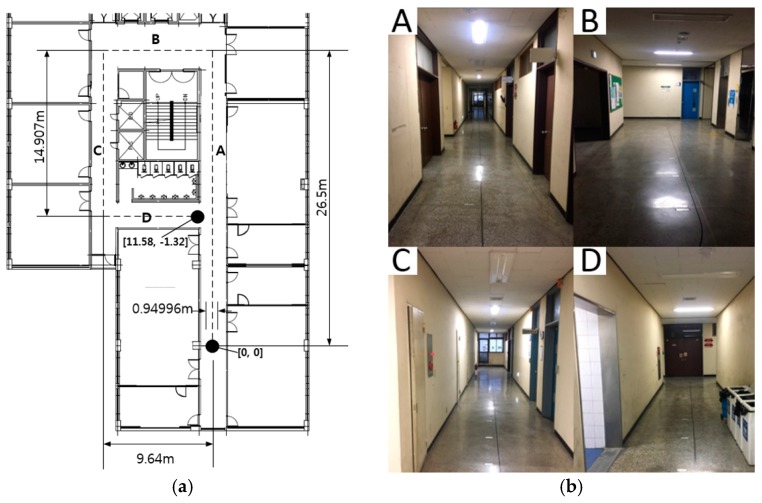
The test area for magnetic field map navigation: (**a**) map of the test area divided into four sections: A, B, C, and D; and (**b**) photos of the test corridor.

The magnetic field map used in this paper was built with magnetic field data from the 7th floor corridor of the 10th engineering building in Pusan National University. This area is shown in Figure 2. The reference system moves from position (0, 0) to position (11.58, −1.32) in the corridor. The magnetic field was measured with 55.88 mm spacing in the direction of mobile robot movement with a width of 0.94996 m (=55.88 mm×17). In order to reduce the error in the magnetic field map, every magnetic sensor was calibrated [13], and the magnetic field was measured several times by changing the position of the sensor. Finally, the magnetic field maps about each axis can be obtained. The magnetic maps created are composed of *X*, *Y*, and *Z*-direction magnetic field maps [13,16,22]. These maps are represented as $M_{X}(r)$, $M_{Y}(r)$, and $M_{Z}(r)$, where $r={\left[r_{x},r_{y}\right]}^{T}$.

If all three magnetic field maps are used for estimating position, the estimation error is reduced remarkably, compared to magnitude magnetic field map $M_{XYZ}(r)=\left\|\left(M_{X}(r),M_{Y}(r),M_{Z}(r)\right)\right\|$. But the amount of computation increases in proportion to the number of magnetic field maps used. If the mobile robot moves on an even floor, the *X*-*Y* magnetic field map, $M_{XY}(r)$, which is the intensity map of the horizontal plane, is used. Then, the amount of calculations can be reduced, compared to using three magnetic field maps. Also, an angle map of the magnetic field, $M_{\varphi }(r)$, is needed. This angle map is not directly used to compute the measurement probability density. It is used to refer to the rotation transformation in this proposed method. This will be explained in detail in the next section. The *X*-*Y* magnetic field map and the angle map are indicated by

(2) $$M_{XY}(r)=\sqrt{{\left(M_{X}(r)\right)}^{2}+{\left(M_{Y}(r)\right)}^{2}}$$

(3) $$M_{\varphi }(r)=\tan^{-1}(M_{Y}(r)/M_{X}(r))$$

**Figure 3:**
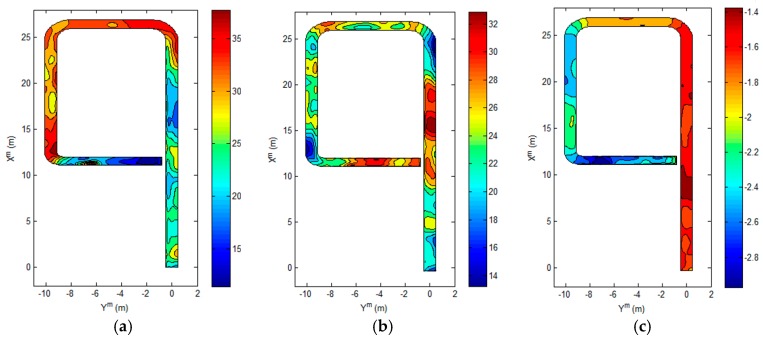
The magnetic field maps. (**a**) The *X*-*Y* magnetic field map, which is the intensity magnetic field map of the horizontal plane; (**b**) *Z*-direction magnetic field map; (**c**) Direction of the magnetic field in the horizontal plane.

The intensity magnetic field maps $M_{XY}(r)$ and $M_{Z}(r)$, and the direction magnetic field map, $M_{\varphi }(r)$, about the test area are shown in Figure 3.

**Figure 4:**
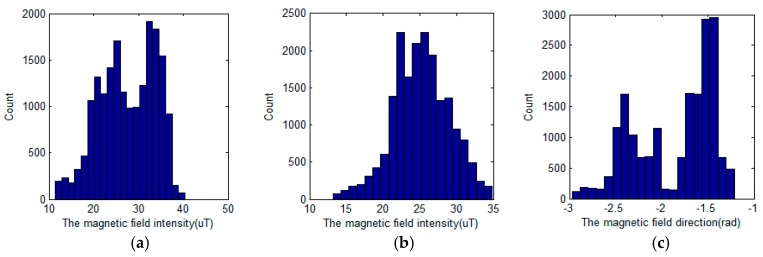
The histograms of the magnetic field maps. (**a**) The *X*-*Y* magnetic field map; (**b**) *Z*-direction magnetic field map; (**c**) Direction magnetic field map in the horizontal plane.

In magnetic field map navigation, the performance of the system depends on variation of the magnetic field maps. The variation of the magnetic field maps can be evaluated by the variance and gradient. If the variance of the magnetic field intensity is larger, it is easy to distinguish between true and false positions. The gradient magnitude of a magnetic field affects the resolution when estimating position. However, if the variance and gradient magnitude of the direction magnetic field map are large, the estimated orientation has a large error. This is explained in detail in the next section. For the above reasons, the variance and gradient magnitude of the magnetic field map at each point are important elements in magnetic field map navigation. Table 1 shows the statistics of the magnetic field maps in the test area. Also, the histograms of the magnetic field maps are shown in Figure 4.

### 2.3. Frame Definition

**Figure 5:**
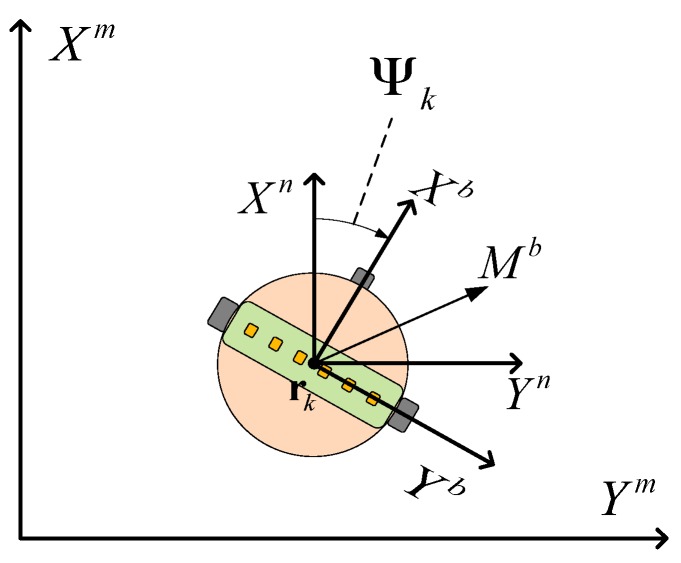
The various frames.

The estimated position is expressed in relation to the main frame in this paper. Since the sensor data are measured in the body frame, axis definitions and transformations are required. Figure 5 shows various frames. The mobile robot position is $r_{k}={\left[r_{x,k},r_{y,k}\right]}^{T}$ at time k in the fixed main frame. The navigation frame is parallel to the main frame, but the center of the navigation frame is at the center of the mobile robot [25]. The body frame is the center of the robot. The *X*-axis of the body frame is the same direction as the head direction of the robot. The *Y*-axis is the direction to the right, and the *Z*-axis is directed down the mobile robot. Angle $\Psi_{k}$ is the difference in the angle between the *X*-axis of the body frame and the *X*-axis of the navigation frame.

### 2.4. Particle Filter

When a mobile robot moves on an even floor where three magnetic field maps have already been acquired, the magnetic field data and the encoder velocity data are measured at constant intervals. And then, the particle filter system estimates the position of the mobile robot whenever the magnetic field data are measured. First, one thousand particles are propagated by measured encoder velocity $v_{k}$ and the statistical information of the system. The distribution of the encoder velocity error is assumed to be Gaussian. The propagation model equation is expressed as:

(4) $${\hat{r}}_{k}^{\left(j\right)}=r_{k-1}^{\left(j\right)}+v_{k}•\Delta t+e^{\left(j\right)}$$

where $e=Ν(0,\delta_{ENC}^{2})•\Delta t$, and *j* is the particle index.

Second, measurement probability density function $p(m_{k}|r_{k})$ that is able to update the weight of a particle has to be evaluated. PDF $p(m_{k}|r_{k})$ presents the outcomes, which include information on the actual position in statistical inference. The magnitude of the outcomes near the actual position is relatively higher than other outcomes. If the distribution of the magnetic field measurement error is Gaussian, the PDF by using one sensor is represented as:

(5) $$p(m|r)=\gamma \exp\left(-\frac{1}{2\delta_{e}^{2}}{\left(m-M(r)\right)}^{2}\right)$$

where $\gamma =\frac{1}{\sqrt{(2\pi )\delta_{e}^{2}}}$ and where m is the magnetic field measurement by the sensor, M(r) is the magnetic field magnitude value at r in the map, and $\delta_{e}^{2}$ is the measurement error variance [16]. In order to consider the orientation of the mobile robot, the direction magnetic field map, $M_{\varphi }(r)$, and the measured direction of the magnetic field in the horizontal plane are used. If the *i*-th magnetic field sensor is placed at $r_{i}^{b}$ in the body frame, which is located at $r_{k}$ and rotated about $\Psi_{k}$ in the main frame, the sensor position in the main frame is expressed as

(6) $$r_{i,k}^{\prime }=Rot(\Psi_{k})•r_{i}^{b}+r_{k}$$

where $Rot(\Psi_{k})$ is rotation matrix about $\Psi_{k}$. The measurement of the magnetic field at $r_{i,k}^{\prime }$ is expressed as

(7) $$m_{i,k}^{b}=\left[\begin{array}{cc} Rot(-\Psi_{k}) & 0\\ 0 & 1 \end{array}\right]\left[\begin{array}{c} M_{X}(r_{i,k}^{\prime })\\ M_{Y}(r_{i,k}^{\prime })\\ M_{Z}(r_{i,k}^{\prime }) \end{array}\right]+Ν(0,\delta_{e}^{2})$$

with the measurement of the *i*-th sensor represented as $m_{i,k}^{b}={\left[m_{i,x,k}^{b},m_{i,y,k}^{b},m_{i,z,k}^{b}\right]}^{T}$. However, to calculate $r_{i,k}^{\prime }$, it is necessary to know the mobile robot’s orientation, $\Psi_{k}$. The orientation can be estimated by using the direction magnetic field map, $M_{\varphi }(r_{k})$, and the direction of the measured magnetic field, $\varphi_{k}^{b}$, at the center of the body frame. The relationship is expressed as follows [26]:

(8) $$\Psi_{k}^{\prime }(r_{k})=M_{\varphi }(r_{k})-\varphi_{k}^{b}$$

In order to overcome the sensor noise, the direction of measured magnetic field $\varphi_{k}^{b}$ is estimated with the mean of the magnetic field direction from each sensor because the sensors are located close each other in the mobile robot. This direction is expressed as:

(9) $${\hat{\varphi }}_{k}^{b}=\frac{1}{n}\sum_{i=1}^{n}\tan^{-1}(m_{i,y,k}^{b}/m_{i,x,k}^{b})$$

where *n* is the number of sensors used, and *i* is the sensor index. However, if the variation of the direction magnetic field map is large, the estimated direction of magnetic field ${\hat{\varphi }}_{k}^{b}$ has a large error, because the directions measured by each magnetic sensor are greatly different from each other. After the mobile robot orientation, $\Psi_{k}^{\prime }(r_{k})$, is computed, $r_{i,k}^{\prime }$ is expressed as:

(10) $$r_{i,k}^{\prime }=Rot(\Psi_{k}^{\prime }(r_{k}))•r_{i}^{b}+r_{k}$$

The two PDFs about the *X*-*Y* magnetic field and Z-direction magnetic field are expressed as:

(11) $$p({m^{\prime }}_{i,xy,k}^{b}|r_{k})\propto \exp\left(-\frac{1}{2\delta_{e,xy}^{2}}{\left({m^{\prime }}_{i,xy,k}^{b}-M_{XY}(r_{i,k}^{\prime })\right)}^{2}\right)$$

(12) $$p({m^{\prime }}_{i,z,k}^{b}|r_{k})\propto \exp\left(-\frac{1}{2\delta_{e,z}^{2}}{\left({m^{\prime }}_{i,z,k}^{b}-M_{Z}(r_{i,k}^{\prime })\right)}^{2}\right)$$

where ${m^{\prime }}_{i,k}^{b}={\left[\sqrt{{\left(m_{i,x,k}^{b}\right)}^{2}+{\left(m_{i,y,k}^{b}\right)}^{2}},m_{i,z,k}^{b}\right]}^{T}={\left[m_{i,xy,k}^{b},m_{i,z,k}^{b}\right]}^{T}$.

If the PDFs about $m_{i,xy,k}^{b}$ and $m_{i,z,k}^{b}$ are assumed to be an independent distribution, the joint PDF is expressed as:

(13) $$p({m^{\prime }}_{i,k}^{b}|r_{k})=\frac{p({m^{\prime }}_{i,xy,k}^{b}|r_{k})p({m^{\prime }}_{i,z,k}^{b}|r_{k})}{C}$$

where *C* is a normalizing constant. Also, if the measurement PDFs of each sensor can be assumed to be an independent distribution, the final joint PDF is represented as:

(14) $$p({m^{\prime }}_{1:n,k}^{b}|r_{k})\propto \prod_{i=1}^{n}p({m^{\prime }}_{i,k}^{b}|r_{k})$$

with ${m^{\prime }}_{1:n,k}^{b}$ ≜ ${m^{\prime }}_{1,k}^{b},\cdots ,{m^{\prime }}_{n,k}^{b}$.

After $p({m^{\prime }}_{1:n,k}^{b}|r_{k})$ is evaluated by measurement, the importance weights of the particles are updated. The measurement update step is expressed as:

(15) $$w_{k}^{\left(j\right)}=w_{k-1}^{\left(j\right)}p({m^{\prime }}_{1:n,k}^{b}|{\hat{r}}_{k}^{\left(j\right)})$$

After the measurement update step, the updated weights of particles are normalized so they sum to 1. Since the particles are normalized, we do not have to calculate the constant *C* in order to reduce processing time.

Finally, the mobile robot position is estimated by means of the particles where the equation is represented as:

(16) $${\hat{r}}_{k}=\sum_{j=1}^{N}{\hat{r}}_{k}^{\left(j\right)}•w_{k}^{\left(j\right)}$$

**Figure 6:**
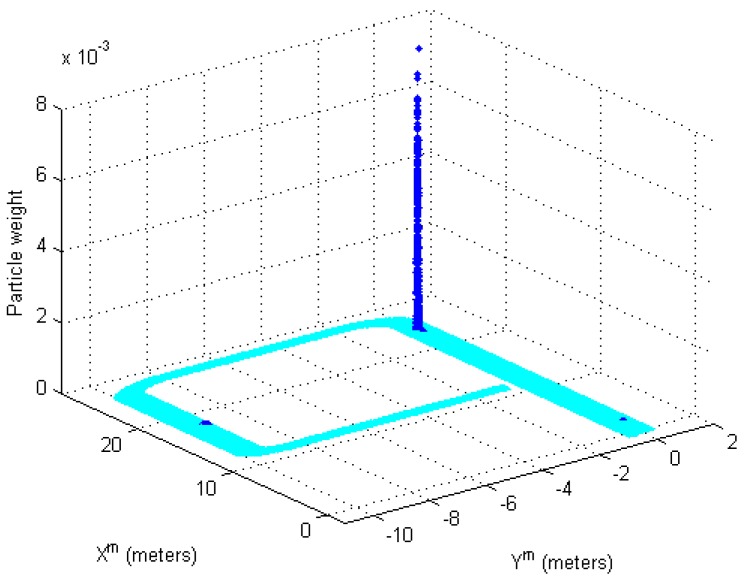
The updated importance weights in the measurement update step.

Figure 6 shows the updated importance weights in the measurement update step. The particles propagated by the encoder in the previous step are updated to a new weight through computation with the measurement PDF. When the mobile robot position is (23.25, 0) in Figure 6, the particles are gathered near (2, 0), (18.34, −9.16), and the real position of the mobile robot. However, since the probability density near the real position is higher than the others, the estimated position of the mobile robot is able to be near the real position of the mobile robot. Using Equation (16), the position is estimated as (23.23, −0.03). Over time, the particles near (2, 0) and (18.34, −9.16) will gradually be cleaned up, because these particles have very low weight.

If the position of the mobile robot can be estimated, the orientation of the mobile robot is also determined. The estimated orientation of the particles is expressed as:

(17) $${\hat{\Psi }}_{k}^{\left(i\right)}=M_{\varphi }({\hat{r}}_{k}^{\left(i\right)})-{\hat{\varphi }}_{k}^{b}$$

Also, the orientation of the mobile robot is estimated with the following equation:

(18) $${\hat{\Psi }}_{k}=\sum_{j=1}^{N}{\hat{\Psi }}_{k}^{\left(j\right)}•w_{k}^{\left(j\right)}$$

**Figure 7:**
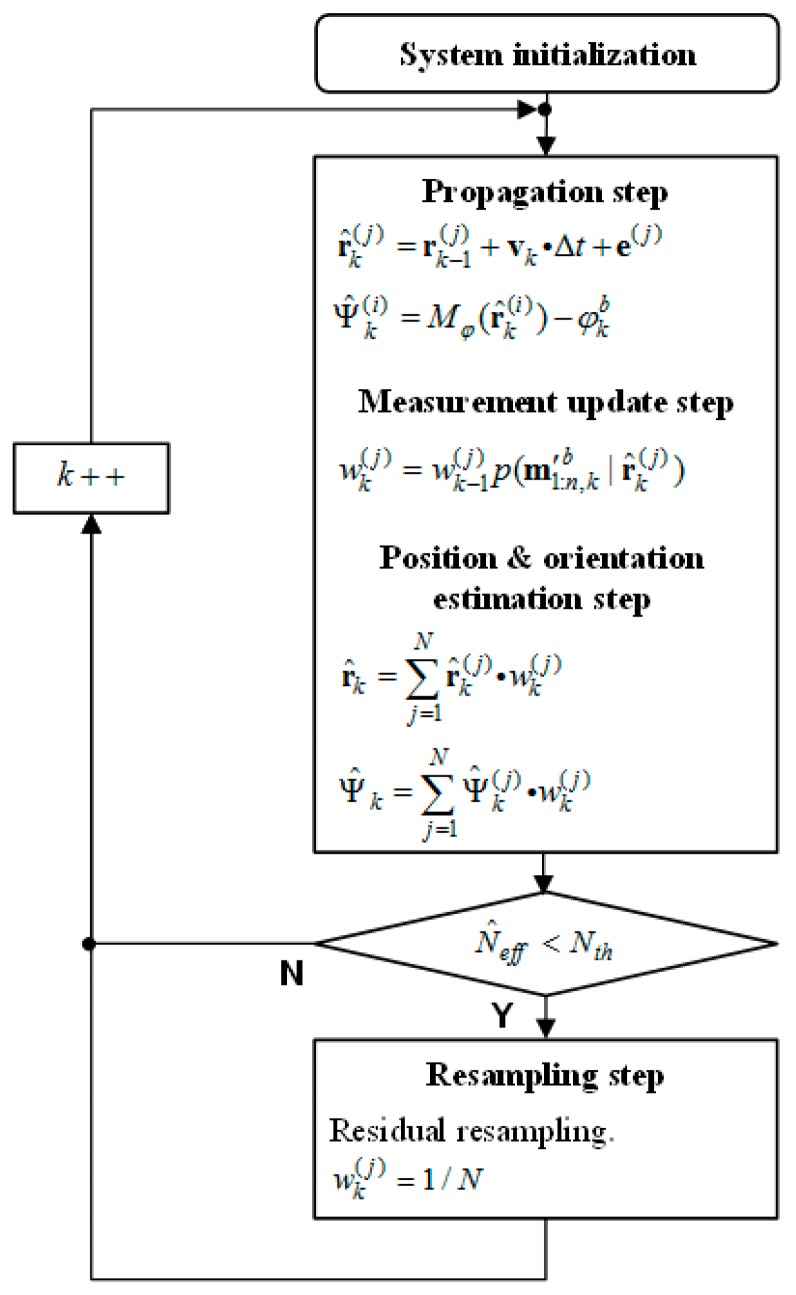
The proposed system flowchart.

In order to avoid the degeneracy problem of importance weights, the resampling step is required. If the effective number of particles, ${\hat{N}}_{eff}$, is smaller than threshold number $N_{th}$, residual resampling is performed. This condition is expressed as

(19) $${\hat{N}}_{eff}=\frac{1}{\sum_{j=1}^{N}{\left(w_{k}^{\left(j\right)}\right)}^{2}}<N_{th}=N/2$$

where *N* is the number of particles, and *N* = 1000 in this system. After the residual resampling, the weight of all particles is equalized to 1/*N*. Figure 7 shows the flowchart of the proposed particle filter system.

## 3. Experiments and Results

### 3.1. Experimental Environment

**Figure 8:**
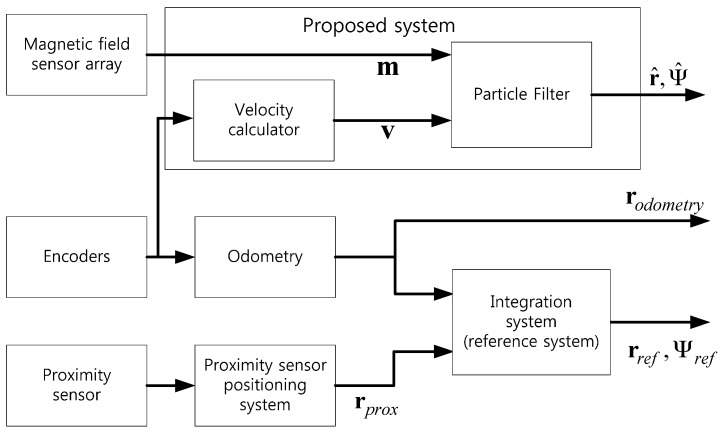
Block diagram of the proposed system and the reference system.

**Figure 9:**
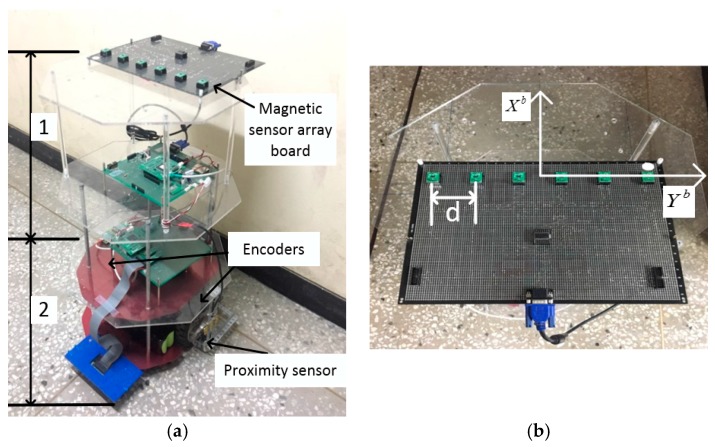
The mobile robot system. (**a**) Section 1 is the measurement and data transmission unit, and Section 2 is the control unit; (**b**) The magnetic sensor array board.

**Figure 10:**
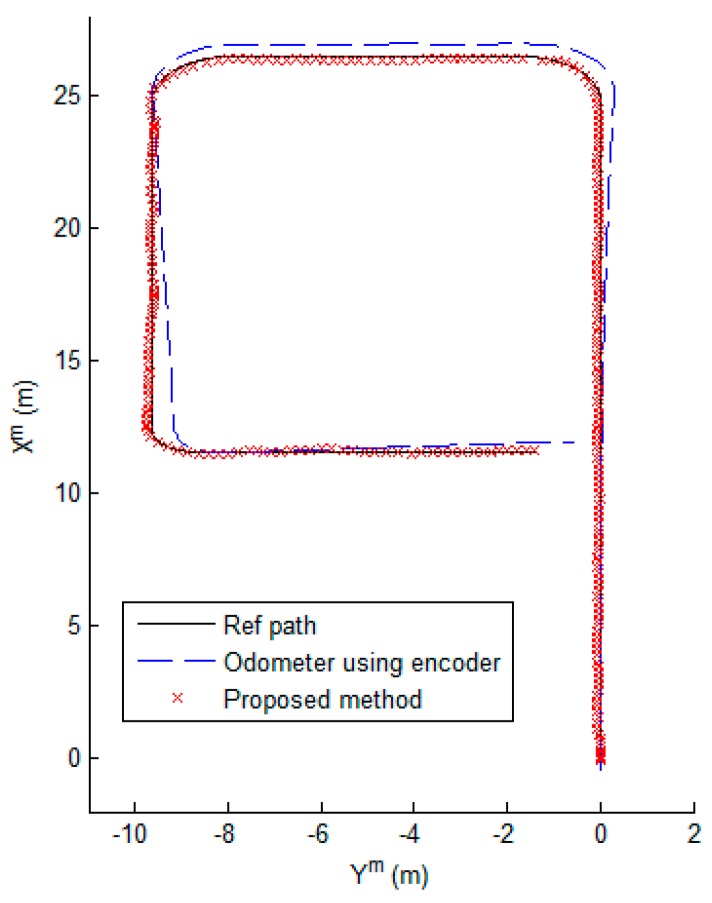
The estimated position of the mobile robot.

In order to evaluate the proposed system, a reference system and odometry using encoders were used. Figure 8 shows a block diagram of these systems. The reference system that was used to build the magnetic field map prints reference position $r_{ref}$ and reference orientation $\Psi_{ref}$. Also, odometry using encoders prints position $r_{odometry}$. Finally, the proposed system estimates position $\hat{r}$ and orientation $\hat{\Psi }$ for the mobile robot. For this paper, we used a Stella B2 mobile robot as a platform to obtain the information of the encoders, the magnetic field data, and the proximity sensor output. This mobile robot, shown in Figure 9, is divided into a control unit and a measurement and data transmission unit. The control unit consists of the two encoders and a line tracking system that controls the mobile robot’s motion. The acquired encoder information is transmitted to the measurement and data transmission unit. The measurement and data transmission unit has a magnetic sensor array board and a proximity sensor. The magnetic sensor array board is composed of six LSM303 magnetic field sensors (STMicroelectronics, Geneva, Switzerland), which are arranged on the $Y^{b}$ axis at intervals of *d* (=55.88 mm), as seen in Figure 10. This unit transmits the received encoder data, the measured magnetic data, and the proximity sensor data to a personal computer (PC).

Figure 2 shows the path of the mobile robot in the corridor. A tracking line was installed along the center of the corridor. The proximity sensor’s landmarks were installed at known positions. The mobile robot moved from (0, 0) to (11.58, −1.32). Before the mobile robot departed, the particles were updated many times. The system was then able to operate stably. When the mobile robot moved along the predetermined path in the corridor, each sensor’s data and the reference data were transmitted periodically. Finally, the proposed algorithm was executed by the PC twice per second.

### 3.2. Results

**Figure 11:**
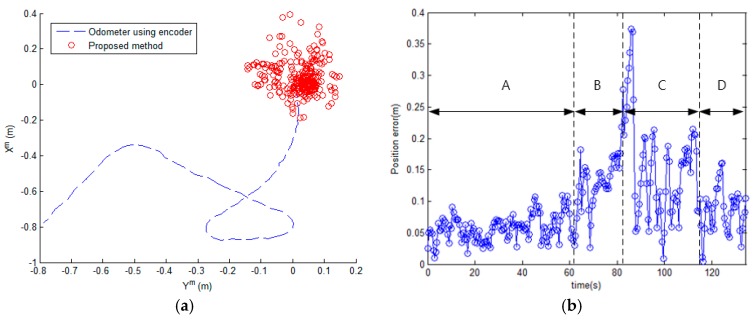
The position error of the mobile robot. (**a**) Comparison of the odometry position errors and the proposed method’s position errors; (**b**) The distance errors of the proposed method. A, B, C, and D are the sections of the test corridor, as shown in Figure 2.

**Figure 12:**
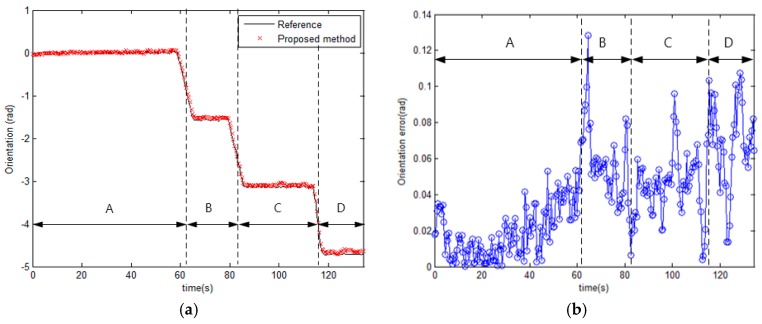
The estimated orientation from the proposed method. A, B, C, and D represent the sections of the test corridor, as shown in Figure 2. (**a**) The estimated orientation and the reference orientation; (**b**) The errors from the estimated orientation.

The results of the proposed method are shown in Figure 10, Figure 11 and Figure 12. Figure 10 shows that the mobile robot position was estimated near the path. However, the estimated position from odometry using the encoders was off the path. Figure 11 shows the position error of the mobile robot. Comparison of the odometry position errors and the proposed method’s position errors are shown in Figure 11a. At the beginning of the experiment, the estimation results from odometry using the encoders was less than with the proposed system, but the estimated position from odometry diverged over time. We can see that the proposed method prevents divergence by the encoder. Figure 11b shows the distance error with the proposed method. The distance error occurs largely in the vicinity of 90 s, passing through the second corner between sections B and C. Also, the variance of the distance error in sections C and D is larger than in section A. Because the variation of the direction in the magnetic field map is large as shown in Figure 3c. In the process of estimating the position, the estimated direction of the magnetic field ${\hat{\varphi }}_{k}^{b}$ is used as shown in Equations (8)–(10). In order to reduce the influence of noise, the magnetic field direction from each sensor is averaged, as shown in Equation (9). However, if the variation of direction is large, the mean of the magnetic field direction ${\hat{\varphi }}_{k}^{b}$ has large error. As a result, the error of estimated position becomes large. In the case of section B, the variation of direction is small, but the distance error is larger than in section A. Because the distance error is also affected by the variation of intensity. As shown in Figure 3a, the variation of X-Y intensity in section B is lower than in section A. If the variation of intensity is small, the variance of the measurement PDF becomes large. Since the weight of particle is updated by the measurement PDF as shown in Equation (15), if the variance of the measurement PDF is large, the estimated position is able to have a large error. Therefore, the smaller the variation of direction and the larger the variation of intensity, the better the performance of the position estimation.

For the same reason, the variations of intensity and direction affect the performance of orientation estimation. Figure 12 shows the estimated orientation of the mobile robot. The orientation error in sections B, C, and D is larger than in section A. If the variation of direction is large, the error of estimated magnetic field direction ${\hat{\varphi }}_{k}^{b}$ becomes large. As a result, the estimated orientation has a large error as shown in Equation (17). Also, the variation of the intensity affects the variance of the measurement PDF, and the weight of particles updated by PDF affects the orientation estimation as shown in Equation (18). If the variation of the intensity is small, the estimated orientation has a large error. Finally, the experimental results show that the smaller the variation of direction and the larger the variation of intensity, the better the performance of the position and orientation estimation. The error statistics of the proposed method are shown in Table 2. The mean distance error is within 0.1 m, and the maximum distance error is about 0.4 m. The mean orientation error is 0.0386 rad, and the maximum error in orientation is approximately 0.13 rad.

**Figure 13:**
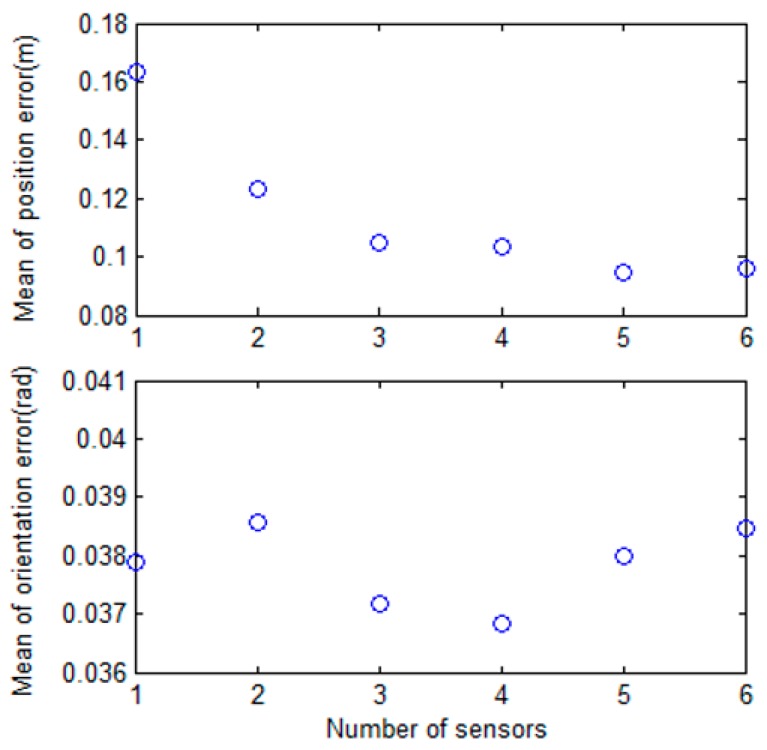
The results depending on the number of sensors used. The upper figure shows the mean of the distance error for the estimated position. The mean of the orientation error is shown in the lower figure.

**Figure 14:**
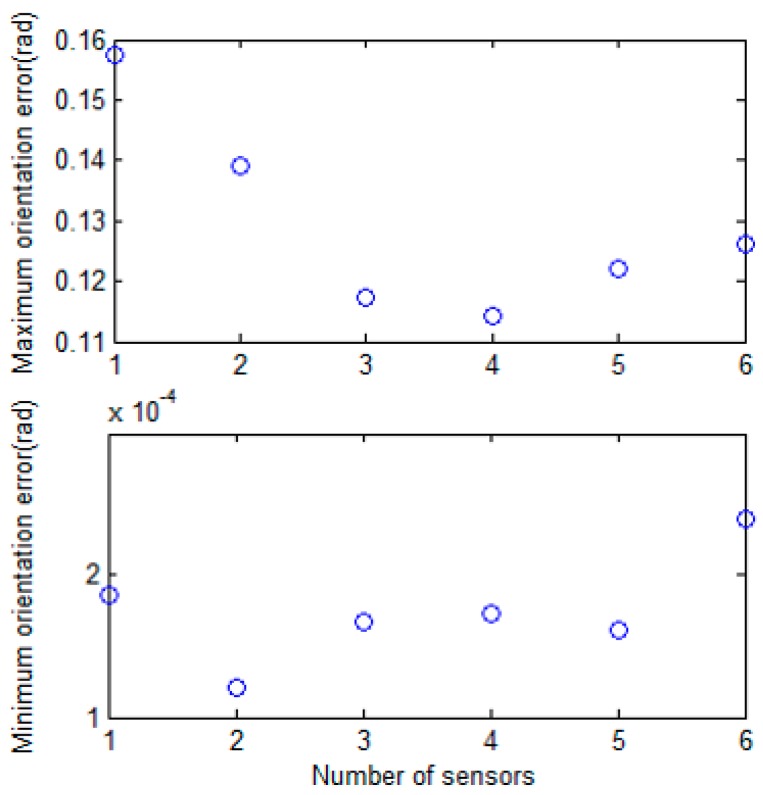
The maximum and minimum orientation error results depending on the number of sensors used.

Figure 13 shows the distance and orientation error results, depending on the number of sensors used. Experiments were performed by increasing the number of sensors from one to six, and each experiment was performed 10 times. For distance error in the estimated position, as the number of sensors increased, the performance improved, and the distance error converged at about 0.1 m. However, when three or more sensors were used, there was little difference in performance. The results of orientation error were similar in the experiment. The maximum and minimum orientation errors are shown in Figure 14. When more sensors are used, the maximum error tends to decrease. Since the processing time is proportional to the number of sensors used, it is reasonable to use three sensors in this system.

## 4. Conclusions

In this paper, we proposed a positioning system using a magnetic field map navigation and an encoder system. Before estimating the position of a mobile robot, a magnetic-field map-building system was implemented to efficiently obtain three magnetic field maps: a horizontal intensity map, a vertical intensity map, and a direction information map. After the three magnetic field maps were built, the position of the mobile robot was estimated by using the particle filter. We show that the particles are propagated by the velocity of the encoder without error correction in the propagation step, and the weights of particles are updated by using the three magnetic field maps and multiple magnetic sensors. As a result of processing with the particle filter, the position of the mobile robot was estimated, and the position estimation performance was better than with odometry. Also, the proposed system estimated the orientation of the mobile robot without help from any other sensor system. As a result of the experiment, we confirmed the relationship between the variation of magnetic field maps and the performance of this system. The smaller the variation of direction and the larger the variation of intensity, the better the performance of the position and orientation estimation. Also, we confirmed that system performance is likely to improve when the number of sensors increases. In this paper, when six magnetic field sensors which are aligned in a line are used, the mean distance error is less than 0.1 m and the mean orientation error is 0.0386 rad.

In order to further improve the performance of the proposed system, the sensors must be calibrated accurately, and the various placement methods for the sensors must also be studied. An integration method with other systems can also be considered, and more research is needed into building a magnetic field map more accurately.

## Figures and Tables

**Table 1:** Statistics of the magnetic field map.

	*XY* (uT)	*Z*-Dir (uT)	Dir (rad)
Std.	6.24	3.83	0.43
Mean	27.55	25.18	−1.86
Max.	40.38	34.84	−1.21
Min.	11.41	13.19	−2.97

**Table 2:** The error statistics of the proposed method.

	Distance (m)	Orientation (rad)
Mean	0.0948	0.0386
Std.	0.0618	0.0310
Max.	0.3736	0.1285
